# Methicillin-Resistant *Staphylococcus aureus* in Pigs and Farm Workers on Conventional and Antibiotic-Free Swine Farms in the USA

**DOI:** 10.1371/journal.pone.0063704

**Published:** 2013-05-07

**Authors:** Tara C. Smith, Wondwossen A. Gebreyes, Melanie J. Abley, Abby L. Harper, Brett M. Forshey, Michael J. Male, H. Wayne Martin, Bayleyegn Z. Molla, Srinand Sreevatsan, Siddhartha Thakur, Madhumathi Thiruvengadam, Peter R. Davies

**Affiliations:** 1 Center for Emerging Infectious Diseases, Department of Epidemiology, University of Iowa College of Public Health, University of Iowa, Iowa City, Iowa, United States of America; 2 Department of Veterinary Preventive Medicine, Ohio State University, Columbus, Ohio, United States of America; 3 Department of Veterinary Population Medicine, College of Veterinary Medicine, University of Minnesota, St. Paul, Minnesota, United States of America; Department of Population Health and Pathobiology, College of Veterinary Medicine, North Carolina State University, Raleigh, North Carolina, United States of America; University of Edinburgh, United Kingdom

## Abstract

Much uncertainty remains about the origin and public health implications of livestock-associated methicillin-resistant Staphylococcus aureus (LA-MRSA). This study aimed to investigate the occurrence and prevalence of MRSA in general and LA-MRSA in particular in pigs and farm workers in five states. We collected nasal swabs from pigs and farm workers at 45 swine herds (21 antibiotic-free herds; 24 conventional herds) in Illinois, Iowa, Minnesota, North Carolina and Ohio. MRSA was isolated from 50 of 1085 pigs (4.6%) and 31 of 148 (20.9%) of farm workers. MRSA-positive pigs and people were clustered in four conventional swine farms in Iowa and Illinois. Based on genotyping, spa type t034, a common livestock associated variant, was predominant among both human and swine isolates. These results confirm the presence of LA-MRSA in pigs and swine farm workers in the USA, but the prevalence found is relatively low compared with European studies.

## Introduction

*Staphylococcus aureus* is a commensal and opportunistic pathogen of man and many homeothermic species. In humans, the clinical manifestations of S. aureus infections vary broadly from minor superficial skin lesions to severe and sometimes fatal invasive disease. Approximately 20–30% of people are colonized with S. aureus, with the most common site for colonization being the anterior nares [1], [2]. While colonization itself does not harm the host, it has been associated with increased risk of developing infections [3]. Both asymptomatic carriers and infected individuals may transmit S. aureus directly or indirectly to others.

Throughout the antimicrobial era, *S. aureus* has displayed great capacity to respond to novel antibiotics by acquisition of resistance determinants, including the rapid emergence of methicillin-resistant strains (MRSA) following the introduction of that drug for medical use in 1961 [4]. On the basis of data from the 2001–2004 National Health and Nutrition Examination Survey (NHANES), it was estimated that 1.5% of the U.S. population (∼4.1 million persons) is colonized with MRSA [1]. Antimicrobial resistance in S. aureus is a major concern in human clinical medicine, and case fatality rates for bacteremic cases remain of the order of 20–40%, with mortality risk in MRSA cases approximately twofold that of cases of methicillin-susceptible S. aureus bacteremia (MSSA) [5], [6].

Before 1995, human MRSA infections occurred predominantly in hospitalized patients who acquired the infections nosocomially. Subsequently, MRSA infections have been reported increasingly in people who had not been hospitalized nor had underlying illnesses. Furthermore, the predominant strains causing healthcare-associated MRSA infections (HA-MRSA) have been distinct from those isolated from community-associated MRSA infections (CA-MRSA) [7].

Although S. aureus is a commensal of several mammalian species, until recently animals were considered of negligible significance as reservoirs for human S. aureus infections. Since 2004 multiple reports of MRSA in animals worldwide, and apparent animal-to-human transmission, have heightened concerns about the risks of animal populations as potential reservoirs of zoonotic MRSA infections [8], [9]. Of particular concern is the high prevalence of ST398 clonal complex strains (termed ‘livestock associated MRSA’, or LA-MRSA) detected from nasal swabs from livestock (particularly pigs and cattle) and livestock workers in several countries [10], [11], [12]. Much uncertainty remains about the origin and public health implications of LA-MRSA, including the use of antibiotics as drivers of resistance development. Given the paucity of information available about these organisms in the US swine industry, we sampled pigs and farm workers on conventional and antibiotic-free swine farms in several US states.

## Materials and Methods

### Farm selection and enrollment

We collected samples from weaned pigs between 6 and 18 weeks of age (target of 24 per farm) and all workers willing to participate on 45 farms in five major swine producing states in the USA that were readily accessible to collaborating groups: Iowa and Illinois (University of Iowa); Ohio and North Carolina (The Ohio State University); and Minnesota (University of Minnesota). Samples were collected between April 2008 and July 2010. The sample size was adequate to detect a positive herd with more than 99% confidence at an expected prevalence of 20% assuming a population size of 1000 animals. The study was designed so that half of the farms sampled by each collaborating group would be conventional confinement farms and half would be farms raising pigs without use of antibiotics (antibiotic-free, “ABF”). Farms were selected by convenience based on willingness of owners to participate. Due to difficulty in recruiting eligible farms, ultimately 45 farms were included in the study: 18 farms in Minnesota (9 conventional, 9 ABF); 18 farms in Iowa-Illinois (9 conventional, 9 ABF); and 9 farms in Ohio-North Carolina (6 conventional; 3 ABF). Where possible, swine selected were not comingled prior to sampling. This was not always possible on the smaller ABF farms. Twenty-four pigs were sampled on 42 of the farms; 22 on one; 25 on one; and 30 on another.

All swine farm workers at each study farm were invited to participate in the study. After giving written informed consent, participants completed a questionnaire providing demographic data and provided swab samples from the nares and oropharynx to determine MRSA colonization status. The survey recorded data on potential risk factors for MRSA colonization, including information about contact with swine and use of personal protective equipment.

### Ethics Statement

The Institutional Review Boards and the Institutional Animal Care and Use Committees of the three participating universities approved the protocols prior to the initiation of the study.

### Sample collection and bacteriology

Using sterile swabs, nasal samples were collected from both nares of weaned pigs. Swab samples were collected from both nares and the oropharynx of farm workers, apart from in Minnesota where only nasal swabbing was conducted. Swabs were transported in Stuart's medium at 4°C to the respective laboratories. Samples were inoculated into 2 mL enrichment broth containing 10 g tryptone/L, 75 g NaCl/L, 10 g mannitol/L and 2.5 g yeast extract/L. After 24 h incubation at 35°C, a loopful of broth was inoculated onto selective MRSA agar plates (BBL CHROMagar MRSA, Becton, Dickinson and Company). These plates were incubated 24–48 hours at 35°C and examined for MRSA. Isolates were confirmed to be S. aureus by examining their appearance on Gram stain, by catalase test, tube coagulase test and a S. aureus latex agglutination assay (Pastorex Staph-plus, Bio-Rad). Presumptive Methicillin resistant strains were identified by testing for the presence of penicillin binding protein 2 (PBP2′) (MRSA latex agglutination test, Oxoid Ltd., Hants, UK). MRSA isolates were stored at −80°C.

### *spa* typing

All MRSA isolates were typed by partial sequencing of the Staphylococcus protein A (spa) gene [13], [14]. PCR amplicons of the spa gene were obtained using SpAF (5′-GAACAACGTAACGGCTTCATCC-3′) and 1517R (5′-GCTTTTGCAATGTCATTTACTG-3′)."). spa types were assigned using both the eGenomics, Inc (www.egenomics.com) and Ridom (http://spaserver.ridom.de) spa servers.

### Statistical analysis

Risk factors were compared for MRSA-positive and MRSA-negative workers on farms with MRSA-positive herds using Fisher's exact test for categorical variables and the Wilcoxon rank sum test for continuous variables. Swine MRSA-positive prevalence rates and associated 95% confidence intervals were adjusted for clustering within herds using generalized linear mixed models in PROC GLIMMIX to better reflect the uncertainty around the point estimates. For evaluating risk factors for MRSA carriage among swine workers, a multivariable logistic regression model was developed using generalized estimating equations in PROC GENMOD with an exchangeable correlation matrix to adjust for correlation within farms. Specifically, we evaluated the effect of length of swine contact (i.e., daily hours of contact) on MRSA carriage along with potential confounders (including age, race, and gender). A final model was selected based on the minimized Quasilikelihood under the Independence model Criterion statistic. Statistical significance was assessed at α = 0.05. All analyses were conducted using SAS version 9.3 (Cary, NC).

## Results

### Weaned pigs

The number of pigs sampled per farm ranged from 22 to 30, but was within one pig of the target sample size (i.e., 23 to 25 pigs) on 43 farms. On one ABF farm, only 22 pigs were present. A total of 50 of the 1085 pigs sampled in the study were culture positive for MRSA (4.6%; adjusted for clustering: 4.1%, 95% CI 1.2%–14.3%), with culture positive pigs clustered on 4 (9%) conventional farms in Iowa and Illinois. No positive herds were detected among the 27 farms sampled in Minnesota, North Carolina, or Ohio, and all pigs sampled on the 21 ABF herds were negative for MRSA. The MRSA prevalence among pigs on conventional farms overall was 8.5% (50/588; adjusted for clustering: 7.9%, 95% CI 2.2%–28.6%). On MRSA-positive farms, the prevalence of positive cultures ranged from 17% (4 of 24) to 100% (30 of 30) of weaned pigs sampled.

### Farm workers

The number of participating workers per farm ranged from zero to 28. Between one and three workers were sampled on the majority of farms (31/45; 69%). The median age of participants was 38 years (range 17–73 years). The majority of participants were male (122; 82.4%), white (121; 81.7%), and recruited from farms in Iowa or Illinois (87; 58.8%). Four (2.7%) reported a skin or soft tissue infection within the prior year, whereas none reported previous diagnosis with a MRSA infection. A total of 31 of 148 farm workers (20.9%) were culture positive for MRSA. Of these, 27 (87%) worked on farms where MRSA was detected among the sampled swine. The vast majority of positive samples (24/31; 77%) were from 40 workers sampled on the two farms with the highest prevalence of MRSA in pigs. As working on a farm with a MRSA-positive herd was by far the greatest risk factor for worker colonization (53% [27/51] positive vs 4% [4/97]; p-value <0.0001), we restricted our analysis of other potential risk factors to workers from MRSA-positive farms (Table 1). The majority of participants (85%; 41/48 [3 missing data]) reported contact with swine within the three days prior to sample collection. In univariate analysis, no significant association was observed between MRSA carriage and demographic characteristics, various indicators of swine exposure intensity, or use of personal protective gear (Table 1), although there was a general trend of lower MRSA carriage among those using personal protective gear. In a multivariable logistic regression model, white race (OR 5.5 [95% CI 2.0 – 15.2]; reference: Hispanic) and more than 7 hours of swine exposure per day (OR 5.2 [95% CI 4.2 – 6.5]; reference: ≤7 hours of swine exposure per day) were associated with increased risk for MRSA carriage.

**Table 1:** Demographic and occupational characteristics of participating workers from farms with MRSA-positive swine.

Variable	MRSA negative N = 24*	MRSA positive N = 27*	p-value^1^
Sex			0.232
Female	5 (21%)	2 (7%)	
Male	19 (79%)	24 (93%)	
Race			0.144
White	12 (50%)	20 (65%)	
Hispanic	11 (46%)	7 (35%)	
American Indian	1 (4%)	0 (0%)	
Median years worked with swine (IQR^2^)	3 (1–13)	5 (1–20)	0.378
Median number of swine (IQR)	2400 (2000–5000)	3200 (1000–5000)	0.679
Median hours of exposure per day (IQR)	7 (3.5–8)	8 (4–9)	0.226
Use eye protection			
Rarely or never	11 (46%)	19 (70%)	0.094
At least sometimes	13 (54%)	8 (30%)	
Use protective mask			
Rarely or never	12 (52%)	17 (65%)	0.394
At least sometimes	11 (48%)	9 (35%)	
Use coveralls			
Rarely or never	2 (10%)	8 (33%)	0.083
At least sometimes	18 (90%)	16 (67%)	
Use washable boots			
Rarely or never	0 (0%)	2 (9%)	0.223
At least sometimes	24 (100%)	20 (91%)	
Use gloves			
Rarely or never	1 (4%)	0 (0%)	0.469
At least sometimes	22 (96%)	26 (100%)	

* Not all categories sum to the column total because of missing data for some participants.

1 p-values are based on Fisher's exact test (for categorical data) or the Wilcoxon rank sum test (for continuous data).

2 Interquartile range (IQR), represented by the values of the 25^th^ and 75^th^ percentiles.

### *spa* typing

A total of 11 spa types were detected from 68 isolates tested (Table 2). t034, a common spa type within the ST398 lineage of LA-MRSA, was the predominant spa type detected among humans and swine (50 of 68 tested; 74%). One farm accounted for 6 of 7 isolates belonging to spa type t002, including 4 isolates from humans and 2 from pigs. On three farms (one each from Iowa [t034], Minnesota [t021], and Ohio [t034]), single human samples were positive for MRSA where all pigs tested culture negative. The Iowa worker was a truck driver who worked on multiple farms; such information is not known regarding the t034-positive worker from Ohio.

**Table 2:** Distribution of *spa* types among human and swine isolates of MRSA.

*spa* type*	Human	Swine	Total
t002	5	2	7
t011#	1	0	1
t021	1	0	1
t034#	20	41	61
t084	1	0	1
t179	1	0	1
t330	1	0	1
t337	0	1	1
t571#	0	1	1
t688	1	0	1
t3446	0	3	3
Non-typeable	0	2	2
Total	31	50	81

* Ridom *spa* type.

# *spa* types of ST398 lineage.

## Discussion

The emergence of LA-MRSA in major food animal species has raised numerous questions and concerns for public health and livestock industries. First identified in The Netherlands around 2004, these organisms have since been found in swine herds in many European countries and also in North America [10], [11], [15]. Based on screening using farm dust samples, the prevalence of positive swine herds varied widely among European countries, from 0% in England, Ireland and Sweden, to over 41% in Germany and 51% in Spain [16]. As found in our study, within positive herds, prevalence of culture positive pigs is typically high (17% to 100% of pigs positive). Initial studies in North America indicated that the prevalence of LA-MRSA in swine may be similar to that reported in Europe. In a convenience sample of 20 herds in Ontario, Khanna et al (2008) found 25% of 285 pigs and 20% (5 of 25) of swine farm workers were culture positive, with spa type t034 predominant [11]. Smith et al (2009) first documented the presence of LA-MRSA in the USA in one of two swine production systems studied, and found a high within-herd prevalence (70% of pigs) in the positive system [10]. Davies et al (in preparation) found LA-MRSA occurring in US swine veterinarians (5%) and in 25% of 539 market hogs from 45 herds slaughtered at major US packing plants, with t034 again being the most common spa type. Given the narrow scope of the study of Smith et al (2009) and the difficulty in interpreting data from market hogs due to the possibility of exposure during transport and lairage, further studies are required to obtain more reliable estimates of the prevalence of MRSA in US livestock industries.

The prevalence found for both farms and pigs in the current study was lower than anticipated from the North American studies, but are very similar to recent data from Canada on market age hogs sampled on farms. Weese et al (2011) found MRSA in 4.6% of pigs on 5 of 46 (11%) of farms. Limited data comparing age groups of pigs suggests that prevalence may be higher in weaned pigs [10], [17], which was why we selected this age group for the current study. Both studies suggest that the prevalence of MRSA in North American pigs may be toward the lower end of the range reported across European countries. Important caveats to this study, like preceding studies, are the limited scope and use of convenience sampling rather than formal random sampling to select herds. For this reason, considerable uncertainty remains about the prevalence of LA-MRSA in the US swine industry. The current study was also deliberately biased by the inclusion of a relatively large proportion of antibiotic-free herds (21/45 herds, 46.7% of the tested farms), and a larger herd level study of pigs on farms across the USA may be warranted.

A substantial obstacle to conducting this study was difficulty in recruiting farms. We attribute this to the unfortunate coincidence of conducting the study in the wake of the 2009 HIN1 influenza pandemic, and the alarmist nature of journalistic coverage of both “swine flu” and LA-MRSA, which together contributed to understandable reluctance by producers to participate. This difficulty may complicate efforts to obtain a random sample of herds in the future.

The observation that LA-MRSA isolated from animals are almost uniformly resistant to tetracyclines (relatively uncommon among common human isolates) focused attention on the role that antimicrobial use, particularly of tetracyclines, in food animals may have had in the emergence of LA-MRSA [17], [18], [19]. Our decision to purposively include both ABF and conventional herds was to obtain preliminary data on MRSA prevalence in these different groups of farms in the USA. It is important to point out that the two groups of farms differ in many respects other than use of antimicrobials, including herd size, sources of genetic stock, and general housing systems. The relatively low prevalence of MRSA we observed among conventional herds confirms that routine antimicrobial use in pigs is not a sufficient cause for emergence of LA-MRSA. Also, the high prevalence of MRSA reported on an ABF herd in Canada suggests that exposure to antimicrobials is also not a necessary condition for the occurrence of LA-MRSA in pigs [17].

LA-MRSA was first detected in Europe in 2004 after several decades of widespread use of tetracyclines and other antimicrobials in food animals, but paradoxically in the aftermath of increased restrictions on in-feed antimicrobial use in several EU countries. Recent studies showing that both MRSA and MSSA isolates of ST398 S. aureus from pigs are resistant to tetracyclines is consistent with the hypothesis that widespread tetracycline use may have resulted in tetracycline resistance in S. aureus in pigs [20], [21], [22], [23]. However it is inconsistent with the suggestion that tetracycline use has been an influential factor in the emergence of LA-MRSA, as MSSA isolates are equally resistant (Aarestrup et al., 2010; Cavaco et al., 2011). In contrast, the concurrent finding of high levels of zinc tolerance in LA-MRSA, but not in LA-MSSA presents an alternative selective factor given the widespread use of zinc as a feed additive to control enteric disease in weaned pigs [22]. However, it is optimistic to expect simple explanations for such complex ecological events.

Our observations on farm workers are consistent with numerous studies demonstrating that people working in close contact with animals colonized with MRSA have a high risk of culture positive nasal swabs [9], [10], [12], [24]. Further confirmation of this phenomenon is unlikely to provide new insight unless accompanied by efforts to understand its biological nature and implications for occupational health. The extent to which culture positive nasal swabs represent transient contamination (as workers are typically sampled in the farm environment) versus ‘true’ colonization (where the organisms are an established component of the human nasopharyngeal microbiota) is unknown. As S. aureus appear be a predominant organism in bioaerosols of swine barns [25] discriminating between transient contamination of airways and true colonization of humans working daily in barns is problematic. In our study, the majority of participants reported swine exposure within three days prior to swab sampling, preventing analysis of longer-term ST398 carriage. It is likely that both contamination and colonization can occur, and recent studies of both research workers and workers on veal farms in The Netherlands indicate the transient contamination may be more common and that regular and continuing exposure to livestock is the major determinant of culture positivity for LA-MRSA [26], [27], [28], [29].

Community based studies in The Netherlands and Germany in regions with high prevalence of LA-MRSA in swine found that risk of culture positivity was largely confined to groups with regular occupational exposure to livestock (farmers and veterinarians) and their immediate families but did not occur in unexposed groups in the communities [8], [24]. Furthermore, although very high prevalence has been described in cross-sectional studies of farm workers, the routine use of multiple enrichment methods to culture samples is likely to result in detection of samples with low numbers. Quantification of LA-MRSA organisms in culture positive farm workers may provide a more meaningful context for evaluating contamination versus colonization events and informing assessment of associated health risks.

Several reports of severe or fatal systemic infections indicate that S. aureus strains of the ST398 lineage have the potential to be serious human pathogens. [30], [31], [32]. Unfortunately, many European reports have not clearly distinguished clinical infections from culture positive swabs from screening [33], and the actual clinical risks associated with livestock exposures and colonization with LA- MRSA are uncertain. Some spa types of ST398 appear to be represented more commonly among human clinical isolates than livestock isolates [34], [35], [36], [37], raising the possibility that different ST398 subtypes may vary in their abilities to colonize and cause disease in humans. Further studies should address the lack of quantitative information about the actual clinical risks associated with livestock exposures and colonization with LA-MRSA.
